# Remedial Hypnotism

**Published:** 1896-10

**Authors:** Ralcy H. Bell


ARTICLE VII.

### REMEDIAL HYPNOTISM.

BY DR. RALCY H. BELL.

Press to my lips the wine of sleep
Sing to my soul, dear, sweet and long—
Dash from my eyes the power to weep
Or lift from my heart the anguish of song.

Man is but a step from the amoeba—which way that step took is not yet certainly known. To call the human race barbarous, even at the dawn of the Twentieth Century, were almost meaningless—so abyssmal and chaotic is the gloom.

Amid all the sounding brass and tinkling cymbals few strains of music come. In the depth of our darkness and murk and feeble are the stars to light us. The mightiest investigators are the sturdiest gropers. Only poets and dreamers soar. And yet a ray of promise is on the mountain tops. To the watcher, slowly the dawn—perhaps the dusk!

What pleases us to call progress may prove a backward stroke. However, as steam is to the old stage so is remedial hypnotism to medical therapeutics.

Hypnotism, in the long night of almost primeval darkness, has its stars and its glow-worms; in this desultory wandering we hope to be guided by the stars; and while we are not forgetful of Charcot and Braid, of Bernheim and Mesmer, in America we look to the great American, Doctor Frederick Henry Gerrish.

Hypnos to the Greek may have meant what sleep means to us—but scientifically speaking, hypnotism affords a world of meaning beyond which the word sleep can in anywise imply.

What hypnotism is, I declare no man knows. Of its peculiarity of manifestation, its actions and reactions through the medium of nerve organism, even into the vegetable functions of life, we have vaguely traced and caught in part. We have heard the surges of an unknown sea, but the mists of mysticism hang thick upon the shore. Waves of subconsciousness beat and break at life's threshold—we feel their potential force, numbly, and yet know nothing of their kinetic energy,

That hypnotism depends upon, or is intimately allied to suggestions, (inspiration or imagination) there can be little doubt.

It would seem that the potency of hypnotism should lie largely in communicative suggestion acting upon nerve centres and ganglia through impulses of ideation, hallucination or illusion and thereby involuntarily upon the various other municipalities of cells in bodily tissues.

As the ways of communicability with the mental zones are many, (even more difficult to understand are the devious and reflex touches upon the smaller and remoter ganglia) so are the courses of suggestion rendered proportionately complex and distressing. Thus it is that the five senses (multiplied by themselves) become, as it were, so many suggestive or hypnotic receivers and transmitters. So only can of "suggestion par attitude" and anto-suggestion. But we cannot stop here; back of "suggestion par attitude" and anto-suggestion—back of all hypnotism runs the course and trend of life. Through all the multifigued web of experience woven of the warp and woof of living change and time we may follow the unbroken threads of heredity and environment. From the period when the first living cell, so to speak, put forth its first attempt at differentiation and speculation, through all the intervening time and types, subtile suggestion has wearily wended. Even fashion is a symbol of suggestion—suggestion materialized maybe. In a thousand ways are we made to see the fascination of suggestion; in a thousand ways may we realize that our system of progressive civilization is after all mostly a serial form of suggestion. Suggestion is childhood's key to education; it is the broad highway to pleasure and the countless paths to pain; it is the power of the drama, the touch of music, in a large measure the potency of the pill from the physician's hand, the pegasus of the poet and the ply of the novelist—in a word: the protaeus-sprite of all human life—the diastatic ferment of the soul.

If we reflect for a moment on the various schemes of religion we cannot well remain oblivious to the awful power of hypnotism or hypnotic suggestion upon the human race. Schemes of religion are schemes of hypnotism; and every religionist is, in greater or less measure, a somnambulist. Charlatanry is responsible for some—the good Buddha, Christ, Mohamet and other powerful hypnotists for the others—millions upon millions of hypnotic automatous, many of whom are cataleptics, as it were; and even Christ and Buddha more remotely responsible than may at first appear.

To enter into detailed reasoning, in proof of such statements which to the casual eye may seem bald and disconnected, is out of the province of this article; a reliance is felt upon the trained mind and scientific trend of the unprejudiced reader for, if not corroborative support, at least honest investigation, for in no other way may truth be wooed and won.

Space forbids even thought of detail. The field of suggestion is the field of metabolic life. It is not confined to nervous matter. Positive and negative hypnotism, post-hypnotic suggestion and Berger's echolali subjects, together with Bjornstrom's groups and divisions relating to hallucinations or allusions, and unilateral hallucinations of touch, taste, smell, hearing, sight and suggestion affecting the muscular and organic senses, must be passed, however reluctantly.

Suffice it to say: nature takes no jumps nor bounds; even optical hallucinations and illusions are governed by the fixed and steadfast laws of optics. And we may be sure that the laws of hypnotism with their concomitant relations and effects are as certainly and precisely natural as any in the universe.

The power of hypnotic suggestion and fascination has been long known among brute trainers. Horses have been hypnotized for so many generations that they are practically born with harness and bit.

It is only when we consider the phenomena of mind reading, thought transference and the transmission of sensations that we confront the apparently miraculous—so incomprehensible, complex and beyond us are these hidden and serpentwise peculiarities.

Remedial Hypnotism :

To begin a citation of cases in which hypnotism was used to remedial ends by scientific gentlemen of standing and repute beyond a shadow of doubt or reproach, would lead to tedious length. For specific information and data the reader is referred to "The Remedial Uses of Hypnotism," by Professor Frederic Henry Gerrish, A. M., M. D., of Portland, Maine. Upwards of five thousand times this brilliant American surgeon has met with success bordering the marvellous.

The methods of hypnotism are almost as numerous as the hypnotizers themselves, nevertheless, the groundwork and principle are ever the same.

To Summarize :

Hypnotic suggestion in the hands of enlightened and conscientious physicians is a remedial means—a beneficial resource—of incalculable good. In the hands of charlatans and the ignorant it becomes a menace to morals, a leverage to crime and a threat at the integrity of society.

To forecast :

Remedial hypnotism is the dawn of a new and better epoch in modern therapeutics.—*Moody's Magazine of Medicine.*

